# Longstanding overt ventriculomegaly in adults (LOVA) with patent aqueduct: surgical outcome and etiopathogenesis of a possibly distinct form of chronic hydrocephalus

**DOI:** 10.1007/s00701-021-04983-0

**Published:** 2021-09-07

**Authors:** Giorgio Palandri, Alessandro Carretta, Emanuele La Corte, Diego Mazzatenta, Alfredo Conti

**Affiliations:** 1 Department of Neurosurgery, IRCCS Istituto Delle Scienze Neurologiche Di Bologna, Via Altura 3, 40139 Bologna, Italy; 2 Department of Biomedical and Neuromotor Sciences (DIBINEM), University of Bologna, Bologna, Italy; 3 Programma Neurochirurgia Ipofisi - Pituitary Unit, IRCCS Istituto Delle Scienze Neurologiche Di Bologna, Bologna, Italy

**Keywords:** Longstanding overt ventriculomegaly in adults (LOVA), Late-onset idiopathic aqueductal stenosis (LIAS), Chronic hydrocephalus, Aqueductal stenosis, Endoscopic third ventriculostomy (ETV), Ventriculoperitoneal shunt (VPS)

## Abstract

**Purpose:**

Longstanding overt ventriculomegaly in adults (LOVA) represents a form of chronic adulthood hydrocephalus with symptomatic manifestation in late adulthood. Based on the patency of the aqueduct, two different subcohorts of LOVA can be distinguished. Surgical treatments of this condition are also debated. Therefore, we analyzed preoperative characteristics and clinical outcome after different surgical treatments in a subgroup of LOVA patients with a patent aqueduct.

**Methods:**

Eighteen LOVA patients with a patent aqueduct consecutively treated at our institution between July 2013 and December 2019 were analyzed for this study. Median age was 70 years. Preoperative radiological and clinical features, surgical procedures (ventriculo-peritoneal shunt or endoscopic third ventriculostomy), and outcomes were collected. Successful outcome was qualitatively defined as an improvement or a halt of progression of the presenting symptoms at follow-up, and quantitatively by changes in mRS and iNPHGS scales.

**Results:**

Twelve patients underwent an ETV as a primary treatment, while 6 underwent VPS. A total of 22.2% of them were lost to follow-up. Median follow-up time was 38 months. Six patients (66.7%) in the ETV cohort achieved a successful outcome after treatment, with a complication rate of 11.1%. Two patients underwent rescue VPS after ETV failure with a good outcome. Four patients (100%) underwent primary VPS and achieved a satisfactory outcome after treatment, with a reported complications rate of 25%.

**Conclusion:**

LOVA with patent aqueduct represents, in our opinion, a distinct clinical form of chronic hydrocephalus. For this subgroup, as well as for other forms of LOVA, ETV remains an acceptable first-line treatment option considering the good results, and the low complication rate, obtained in those patients and the hypothesis that hydrocephalus is due to an “intracisternal” obstruction.

## Introduction

Longstanding overt ventriculomegaly in adults (LOVA) represents a form of a chronic adulthood hydrocephalus with a subclinical onset in early childhood, before the closure of the cranial sutures, slowly progressing until decompensation and symptomatic manifestation in late adulthood. It has been first described as a separate pathological entity by Shizuo Oi et al. in 1996 [22]. Based on their work [22][21, 23], the cornerstones of LOVA diagnosis are as follows: “overt supratentorial ventriculomegaly with effacement of the cortical sulci, macrocephaly with a symptomatologic spectrum consistent with chronic hydrocephalus (gait disturbances, urinary incontinence, cognitive impairment, parkinsonism, apathy, subnormal IQ) and radiological evidence of sellar anatomy alterations as an indirect sign of the longstanding course of the disease (although not mandatory).”

However, despite this entity being described over 25 years ago, there is no consensus in the literature about the pathophysiology of LOVA. Oi considered a complete aqueductal stenosis as the primary factor starting the long and insidious LOVA clinical course, while not describing this as a mandatory feature for LOVA diagnosis. Nevertheless, other authors have discarded this hypothesis [14, 25]. Indeed, a complete aqueductal stenosis seems to identify a separate subtype of adult hydrocephalus, described as late-onset idiopathic aqueductal stenosis (LIAS) [6].

The correct surgical treatment for LOVA is also disputed. Some authors, such as Oi and Ved [23, 26], claim endoscopic third ventriculostomy (ETV) as the first treatment option for LOVA. Others [14], in the view that LOVA represents a form of communicating hydrocephalus, proposed a primary role of cerebrospinal fluid (CSF) diversion surgery (i.e., ventriculoperitoneal shunt (VPS)).

It appears that, based on the patency of the aqueduct, two different clinical entities both currently classified as LOVA, can be distinguished. The clinical and radiological features of those two groups, as well as the best treatment modalities, remain poorly investigated [9].

In this study, we selected a subgroup of LOVA patients with patent aqueduct and analyzed their clinical and radiological characteristics. We also report the results of the treatment and make some inferences on the pathophysiology of this specific pathological condition.

## Methods and materials

### Study design and setting

Clinical and surgical records of all patients treated surgically for LOVA with patent aqueduct at our institution between July 2013 and December 2019 (78 months) were retrospectively reviewed. All the surgical procedures were performed by the same senior surgeon (G.P.).

### Data sources

Clinical and treatment data were retrospectively collected in a digital anonymized archive. Follow-up information was obtained by outpatient clinical evaluation or telephone interviews at defined time intervals.

### Definitions

#### Inclusion criteria

The following criteria were required to be included in the study: (1) age > 18 years; (2) clinical and radiological characteristics compatible with the diagnosis of LOVA according to Ved’s criteria (Table 1) [26]; (3) evidence of CSF flow through aqueduct in phase-contrast (PC)–MR sequences with turbulence void signal in T2-weighted images; (4) no previous neurosurgical procedures; (5) availability of complete preoperative and follow-up imaging and clinical data.

**Table 1:** Clinical and radiological criteria used to confirm the diagnosis of LOVA (from Ved et al. [26])

1. Clinical symptoms of hydrocephalus developing in adulthood—e.g., headaches, cognitive decline, imbalance, gait disturbance, psychological disturbance, visual deterioration/diplopia
2. Macrocephaly defined by head circumference > 98th percentile in adulthood (male 53.8 cm; female 52.9 cm);
3. Overt tri-ventriculomegaly (lateral and third ventricles) on neuroimaging, with cortical sulcal effacement and/or destruction of the sella turcica as evidence of longstanding ventriculomegaly
4. Absence of a secondary cause for aqueductal stenosis in adulthood (e.g., previous meningitis, subarachnoid hemorrhage)

#### Exclusion criteria

The exclusion criteria were as follows: (1) any form of secondary hydrocephalus, including post-traumatic, post-infective, post-hemorrhagic, or caused by ab extrinseco compression of CSF pathways from a space-occupying lesion; (2) iNPH patients who were recruited from an ad hoc prospective observational study (the Bologna PRO-HYDRO study). In our institution, all the patients referred for the investigation and treatment of chronic primary adulthood hydrocephalus should undergo specific and precise brain imaging and clinical evaluations from neurologists and neurosurgeons. Radiological data, clinical evidence, tap test response, and comorbidities are subsequently revised by a multidisciplinary board of specialized neurosurgeons, neurologists, neuroradiologists, physiatrists, and anesthesiologists to establish eligible patients for VPS placement [24]. All operated patients met the criteria of “probable iNPH” based on the 2005 international guidelines [20].

#### Variables

The following parameters were retrospectively collected: (1) demographics; (2) surgical procedure data (ETV or VPS); (3) symptoms at admission with particular focus on the presence of symptoms ascribable to Hakim triad and/or of increased intracranial pressure (headache, nausea, papilledema, gait disturbance, urinary incontinence, cognitive impairment); (4) clinical data, including cranial circumference (measured in centimeters), modified Rankin Scale (mRS), and iNPH Grading Scale (iNPHGS) reported in Table 2 [15]; (5) neuroradiological features and in particular evidence of sellar bone distortion, empty sella, DESH sign [18], bulging third ventricle floor (a sign often found in aqueductal stenosis and obstructive hydrocephalus) [8, 19], an enlarged cisterna magna measuring 10 mm or more on midsagittal images [3], Evans’ index, third ventricle width (measured in millimeters), callosal angle and tentorial angle; (6) clinical course after the procedure, complications classified according to Clavien-Dindo grading [5], mRS, iNPHGS, and date of the last follow-up evaluation.

**Table 2:** iNPH Grading Scale (iNPHGS), according to Kubo et al., used in our analysis to determine a clinical improvement at follow-up when compared to preoperative status. The grades in each domain are added up to obtain a single score [16]

Grade	Definition
*Cognitive impairment*	
0	Normal
1	Complaints of amnesia or inattention but no objective memory and attentional impairment
2	Existence of amnesia or inattention but no disorientation of time and place
3	Existence of disorientation of time and place but conversation is possible
4	Disorientation for the situation or meaningful conversation impossible
*Gait disturbance*	
0	Normal
1	Complaints of dizziness of drift and dysbasia but no objective gait disturbance
2	Unstable but independent gait
3	Walking with any support
4	Walking not possible
*Urinary disturbance*	
0	Normal
1	Pollakiuria or urinary urgency
2	Occasional urinary incontinence (1–3 or more times per week but less than once per day)
3	Continuous urinary incontinence (1 or more times per day)
4	Bladder function is almost or completely deficient

### Assessment of outcome and quantitative variables

The collected clinical-radiological data were compared to established criteria available in the literature for LOVA diagnosis, both according to Ved et al. [26] and to those originally proposed by Oi et al. [23].

A successful outcome was qualitatively defined as an improvement or a halt of progression of the presenting symptoms, and quantitatively by at least the reduction of one point in mRS and two points in iNPHGS between the preoperative baseline status and the last follow-up visit. Preoperative and follow-up mRS and iNPHGS were retrospectively collected from patient files, in which their clinical status is comprehensively registered. Moreover, in patients treated with ETV, the presence of flow artifact through the surgical fenestration visible in midsagittal T2-weighted follow-up magnetic resonance imaging (MRI) was also required to define a successful outcome.

The qualitative radiological parameters were independently evaluated by three blinded researchers (A.C, E.L.C. and G.P.), and discussion was performed in case of disagreement. For continuous data collected, the mean among the three measurements was calculated and considered for statistical purposes. Clinical and neuroradiological data were entered into an ad hoc database for statistical analysis.

Data were reported according to the STROBE guidelines for observational studies.

### Surgical technique

In our institution, ETV procedures were performed in a standardized fashion. Through a single burr hole on Kocher’s point, a rigid endoscope was introduced in the lateral ventricle and foramen of Monro until the third ventricle was reached. Then, the floor was fenestrated and enlarged with a 3- or 4-Fogarty balloon. Any secondary membrane found in the prepontine cistern was fenestrated as well [26].

Similarly, VPS procedures were also performed in a standardized fashion. In one case, a Strata® adjustable pressure valve (Medtronic, Inc. Minneapolis, MN, USA) was used. In all the other patients, CODMAN® HAKIM® adjustable pressure valves (Integra LifeSciences Production Corporation. Mansfield, MA, USA) were used, and in two cases with also SIPHONGUARD® (Integra LifeSciences Production Corporation. Mansfield, MA, USA) antisiphon device (ASD) implanted.

### Statistical methods

The statistical analysis was performed with IBM SPSS Statistics Subscription (IBM Corp. Released 2018. IBM SPSS Statistics for Mac. Armonk, NY: IBM Corp.)

To assess the homogeneity of the ETV and VPS cohorts analyzed, continuous preoperative variables (age, cranial circumference, tentorial angle, callosal angle, Evans’ index, third ventricle width, mRS, and iNPHGS) were compared with the Mann–Whitney *U* test. Similarly, categorical variables (sellar bone distortion, empty sella, bulging of third ventricle floor, and all the clinical manifestations) were cataloged in contingency tables according to the patients’ cohort and compared with the Pearson chi-square test. Preoperative and postoperative clinical scores were also compared, as continuous variables, with the Mann–Whitney *U* test to determine a significant improvement.

The *p* value was assumed to be statistically significant when ≤ 0.05.

## Results

### Demographic characteristics

A total of 192 patients consecutively treated at our institution in the selected period were considered and analyzed for this study. Of the total sample, 58 patients were treated for secondary hydrocephalus, 7 patients underwent previous neurosurgical procedures, 23 patients had MR evidence of complete absence of CSF flow through the aqueduct, 64 patients received a diagnosis of iNPH, and 22 patients lacked complete clinical and neuroradiological data.

Eighteen patients met our inclusion criteria. Twelve were males (66.7%) and the male/female ratio was 2:1. The median age was 70 years (interquartile range 64–72).

All the patients’ clinical and radiological parameters are consistent with a LOVA diagnosis according to Ved et al., previously stated as an inclusion criterion [26], but only 10 according to Oi et al. [23].

### Clinical and radiological features

Most patients presented Hakim’s triad, either complete (11 patients, 61.1%) or incomplete (6 patients, 33.3%), but invariably presented gait abnormalities. In only one patient (5.6%), the youngest of our cohort, no Hakim’s triad symptoms but only nausea and headache were reported. Three patients (16.7%) showed symptoms of raised ICP.

Sellar abnormalities, either a distortion in sellar bone or an empty sella, were observed in 16 patients (88.9%) being an almost constant feature of our cohort. Surprisingly, all the patients showed an enlarged cisterna magna.

Univariate analysis did not disclose any significant differences between the preoperative radiological and symptomatological features of the two subcohorts, as described in Table 3.

**Table 3:** Preoperative clinical and radiological features of LOVA patients in our cohort. Data are reported as *n* (%) or median (interquartile range). The rightmost column shows the statistical significance of the comparison of the ETV and VPS subcohorts at univariate analysis

	Total (18 patients)	ETV (12 patients)	VPS (6 patients)	*p*
Age, years	70 (64–72)	68 (63–71)	74 (68.3–75)	0.07
Males	12 (66.7%)	9 (75%)	3 (50%)	0.29
Cranial circumference, cm	58.5 (57–59.75)	58 (57–59)	60 (57.5–60.8)	0.51
Sellar bone distortion	5 (27.8%)	4 (33.3%)	1 (16.7%)	0.48
Empty sella	14 (77.8%)	9 (75%)	5 (83.3%)	0.69
Bulging third ventricle floor	7 (38.9%)	5 (41.7%)	2 (33.3%)	0.73
Mega cisterna magna	18 (100%)	12 (100%)	6 (100%)	1
DESH	2 (11.1%)	1 (8.3%)	1 (16.7%)	0.6
Tentorial angle, °	50 (48–56.5)	49 (47.8–55.5)	51 (50.3–57.8)	0.3
Callosal angle, °	63 (51–80)	59 (49–66)	79 (59.8–88.5)	0.26
Evans’ index	0.44 (0.4–0.48)	0.46 (0.42–0.48)	0.42 (0.39–0.46)	0.18
Third ventricle width, mm	18.5 (14.3–22.8)	21 (15.2–25)	15 ( 12.5–19.6)	0.11
Headache	3 (16.7%)	1 (8.3%)	2 (33.3%)	0.18
Nausea and vomiting	2 (11.1%)	1 (8.3%)	1 (16.7%)	0.6
Gait disturbances	17 (94.4%)	11 (91.7%)	6 (100%)	0.47
Sphyncter abnormalities	15 (83.3%)	9 (75%)	6 (100%)	0.18
Cognitive impairment	12 (66.7%)	7 (58.3%)	5 (83.3%)	0.29

### Clinical course and surgical outcome

All outcome data are summarized in Table 4. Twelve out of 18 patients underwent an ETV as a primary treatment, while 6 underwent a VPS. The treatment, due to the previously described uncertainty about the superiority of a treatment against the other one, was determined according to surgeon’s and patient’s choice, clearly informed about the risks and benefits of the proposed procedures.

**Table 4:** Clinical course of surgically treated LOVA patients in our cohort

No	Procedure	F/U (months)	Preop mRS	F/U mRS	Preop iNPHGS	F/U iNPHGS	Satisfactory outcome	Clinical course	Complications	CDG
1	ETV	40	2	1	5	1	Yes	Unremarkable	None	0
2	ETV	Not available								
3	ETV	82	4	2	3	2	No	VPS after 2 years for radiological evidence of reduced ventriculostomy flow	None	0
4	ETV	18	1	0	0	0	Yes	Unremarkable	Brain abscess	2
5	ETV	Not available								
6	ETV	67	4	4	11	11	No	Clinical deterioration after 1 year of improvement, stable after VPS	Distal catheter revision	3b
7	ETV	7	4	2	7	2	Yes	Unremarkable	None	0
8	ETV	8	3	0	2	0	Yes	Unremarkable	None	0
9	ETV	Not available								
10	ETV	13	4	2	7	3	Yes	Unremarkable	None	0
11	ETV	38	2	1	5	2	Yes	Unremarkable	None	0
12	ETV	23	3	4	5	5	No	Clinical deterioration after hospital admittance for systemic sepsis	None	0
13	VPS	70	2	1	6	3	Yes	Unremarkable	None	0
14	VPS	15	4	4	9	7	Yes	Unremarkable	None	0
15	VPS	72	4	2	8	2	Yes	Unremarkable	Distal catheter revision	3b
16	VPS	Not available						Died after 3 years for unrelated causes	Hemorrhage, epilepsy	2
17	VPS	38	4	0	4	1	Yes	Unremarkable	None	0
18	VPS	Not available								

Four patients (22.2%), three who underwent ETV and one who underwent VPS, did not ever refer to our institution for outpatient follow-up visits or never answered to telephone interviews and, therefore, have been excluded from outcome analysis. Another patient (#16) which underwent VPS died because of unrelated causes (community-acquired pneumonia) 3 years after the surgical procedure. Thus, nine ETV patients and four VPS patients were included in our analysis. Median follow–up time was 38 months (interquartile range 15–67).

Six patients (66.7%) who underwent primary ETV cohort achieved a satisfactory outcome, with a surgical complications rate of 11.1%. Among the three patients who experienced an unsatisfactory outcome, two of them required rescue VPS after ETV failure. The first one (#3) underwent VPS because of the evidence of a significantly reduced CSF flow through the ventriculostomy at follow-up imaging, with an unremarkable clinical course and an overall clinical picture. The other one (#6) underwent VPS after subtle clinical deterioration after almost 1 year of improvement. The procedure halted the symptomatologic progression but failed to achieve an improvement. Moreover, a distal catheter migration requiring an additional surgical procedure was reported. Nevertheless, this patient had many comorbidities (obesity, obstructive sleep apnea syndrome, and ischemic heart disease) which could have likely concurred to the overall unsatisfactory outcome. The last one (#12) experienced clinical deterioration after a prolonged hospitalization for unrelated causes (urosepsis).

All the patients who underwent ETV reported patency of the stoma at follow-up MRI, with only one exception, previously mentioned.

On the other hand, 4 patients (100%) in the analyzed VPS cohort achieved a satisfactory outcome after their primary treatment, with a reported complication rate of 25%. A patient (#15) with a distal catheter migration requiring an additional surgical procedure was reported. Moreover, the same complication was recorded in a patient excluded from our analysis (#16) for the impossibility to obtain a follow-up evaluation.

Mean preoperative mRS and iNPHGS were 3.16 ± 1.07 and 5.53 ± 2.96, respectively. Likewise, mean postoperative mRS and iNPHGS were 1.77 ± 1.48 and 3 ± 3.09, respectively. A significant overall improvement in both mRS (*p* = 0.02) and iNPHGS (*p* = 0.03) at follow-up was reported.

Univariate analysis did not show any significant difference between the preoperative mRS and iNPHGS between ETV and VPS cohorts (*p* = 0.4 and *p* = 0.28). Similarly, the univariate analysis did not show any differences in both postoperative mRS and iNPHGS between the two treatments (*p* = 0.94 and *p* = 0.58).

## Discussion

### Definition of LOVA

LOVA has been firstly mentioned as a separate entity in 1996 by Oi et al. Authors observed this condition in a long-term follow-up of patients diagnosed with spina bifida at an early age, while not inferring any causative role of spinal dysraphism [22]. The same authors further analyzed the disease [21] and in their seminal paper [23] proposed three diagnostic criteria: (1) supratentorial ventriculomegaly; (2) a clinical picture characterized by macrocephaly (above the 98th percentile), subnormal IQ, headaches, parkinsonism, apathetic consciousness, and Hakim’s triad; (3) enlarged/destroyed sella turcica (although not mandatory). The 98th percentile established as a cutoff for macrocephaly was 57 cm for females and 58 cm for males. This strict measurement has been later amended by Ved et al. in 2017 and 98th percentile cranial circumference was set in 53.8 cm in males and 52.9 cm in females, according to the WHO Child Growth Standards (Table 1) [28].

According to Oi et al. [23], aqueductal stenosis is the cause of chronic hydrocephalus in LOVA. Nevertheless, according to some observations by Rekate [25], aqueductal stenosis may be secondary to the third ventricle distortion, thus explaining the unsatisfactory outcome of ETV in his six-patient case series. Moreover, he reports also a case of “opening” of a previous aqueductal stenosis during the follow-up imaging after an ETV. The author proposes that venous sinuses stenosis may play a role in the pathophysiology of LOVA, causing a CSF “back-pressure.” Furthermore, Kiefer et al. in 2005, diverging from the original pathophysiological description by Oi et al., considered the absence of complete obstruction of the aqueduct a basic requirement for the development of LOVA [14]. Thus, it is possible that a substenotic aqueduct allows CSF to flow, but in reduced quantity, and trigger a compensation mechanism that gives a slow, almost asymptomatic course until it will be exhausted. More recently, Ibanez-Botella et al. suggested the presence of “communicating” and “noncommunicating” LOVA and proposed an algorithm for the treatment, also taking into account Rekate’s considerations concerning venous sinuses. According to their findings, an MR angiogram should be performed as a part of the diagnostic algorithm of LOVA, to investigate a possible venous sinus stenosis as a cause of “open-aqueduct” hydrocephalus arising from a longstanding impairment of CSF reabsorption [9].

Accordingly, it is reasonable to consider that there are two different forms of LOVA, as stated by Ibanez–Botella et al. [9]. The first one with complete aqueductal stenosis, which shares many clinical and radiological features with late-onset idiopathic aqueductal stenosis (LIAS) [6, 16], therefore making the achievement of a correct differential diagnosis and classification subtle and difficult, and the second one with at least partially patent aqueduct. We selected this second subgroup of patients, for whom the best treatment modality (ETV vs. VPS) is, as a matter of fact, more difficult to determine.

### Clinical and radiological characteristics

As reported in previous studies [14, 23, 25, 26], the clinical onset of LOVA could occur at any age of adulthood, mainly after the fourth and the fifth decades. This feature clearly reflects the “longstanding” clinical course of LOVA, starting from early childhood before the closure of cranial sutures (therefore allowing the skull to abnormally grow and resulting in macrocephaly in adult life) and insidiously passing in adulthood with late decompensation [25]. Our cohort confirms these results, with a median age of 70, although showing a later clinical onset when compared to previous studies.

The term “overt ventriculomegaly,” as firstly noted by Ibanez–Botella et al., was never clearly defined in previous studies [9]. The severity of ventriculomegaly, and consequently the radiological parameters measuring it (Evans’ index and third ventricle width), should be of a higher degree when compared to the other forms of hydrocephalus, also reflecting the aforementioned “longstanding” course of LOVA and the induced adaptive changes of the brain [27]. For this purpose, the authors proposed a cutoff of Evans’ index of 0.4 to achieve a LOVA diagnosis, instead of the conventional 0.3 identifying ventriculomegaly. A similar result was reported in our study, with a median Evans’ index of 0.44. On the other hand, an Evans’ index of 0.4 or more would have ruled out exactly ¼ of patients, as shown by the interquartile range (Table 3); therefore, such a strict cutoff value should be very carefully analyzed before being established as a standard practice.

**Fig. 1:**
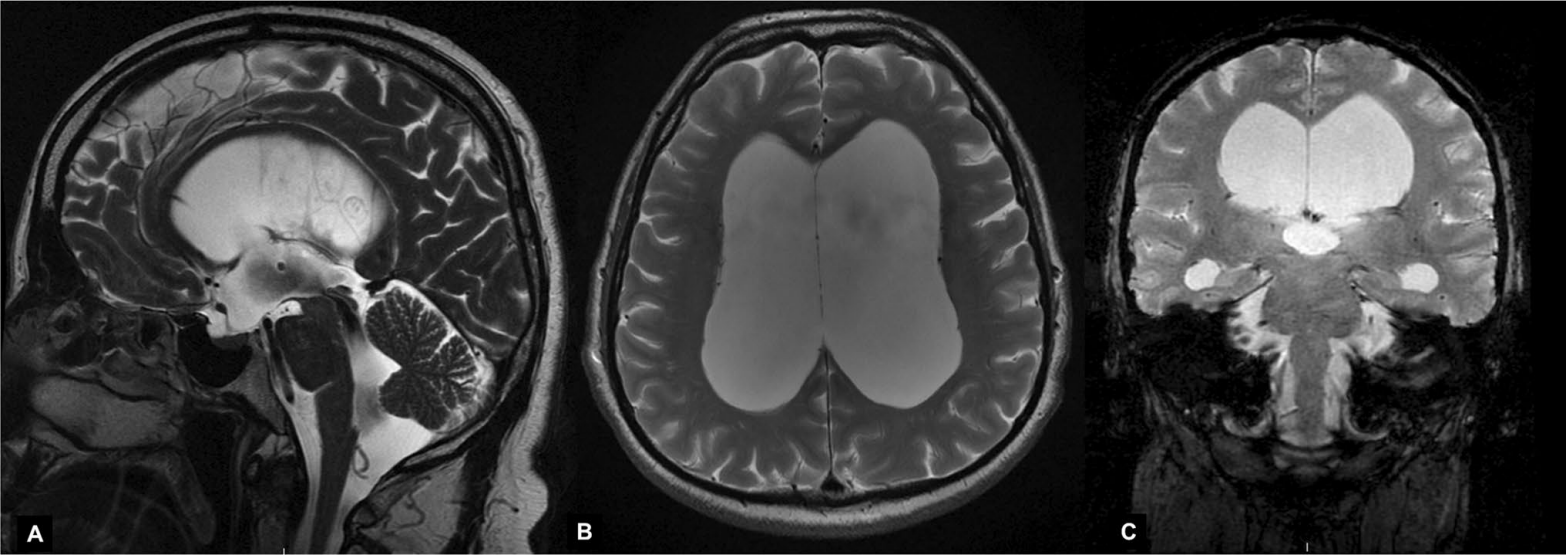
T2-weighted MRI showing peculiar features of LOVA in midsagittal (**A**), axial (**B**), and coronal view. **A** Distorted sellar bone, bulging of the third ventricle floor, patent aqueduct with turbulent flow, and an enlarged cisterna magna are observed. **B**, **C** A concomitant severe ventriculomegaly is reported. Evans index: 0.48. Third ventricle width: 21 mm

**Fig. 2:**
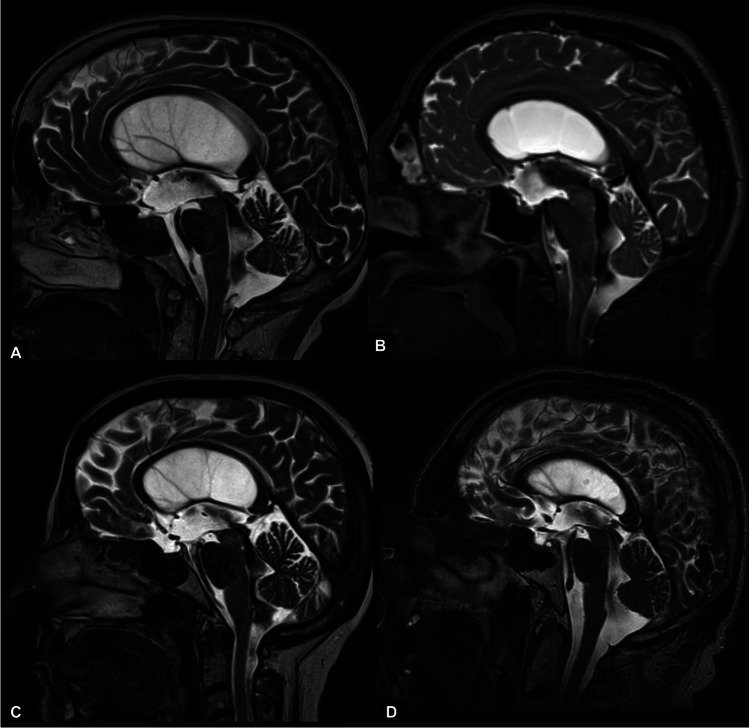
Examples of LOVA in T2-weighted MRI midsagittal view. An enlarged cisterna magna with a patent aqueduct and a turbulent flow is a constant finding (**A**–**D**), among other common peculiar features, such as sellar distortion (**A**, **B**) and third ventricle floor bulging (**A**–**C**). The anatomy of the posterior cranial fossa is distorted as an effect of the cisterna magna enlargement: the folia of the cerebellar vermis are less appreciable and shifted upwards

A relevant finding of our study is the prevalence of enlarged cisterna magna, reported in all patients. Almost invariably, phase-contrast MRI showed a continuous turbulent flow starting from the third ventricle, throughout the aqueduct and fourth ventricle until the cisterna magna. Moreover, distorted anatomy of the posterior fossa could be observed, characterized by an upwards shifted cerebellar vermis, with compressed folia (Figs. 1 and 2).

To our knowledge, this is the first report of the correlation between supratentorial adult hydrocephalus and enlarged cisterna magna. In our opinion, the aforementioned abnormality of CSF flow distally from the fourth ventricle and in the posterior cranial fossa found in LOVA patients, with a concomitant patent aqueduct, plays a role in the pathophysiology of this disease. Some degree of CSF flow obstruction seems to occur at this level leading to a chronically elevated ICP that still allows some neuroplasticity changes to compensate for it [2, 7]. The abnormal anatomy found in this cohort (Figs. 1 and 2) suggests an increased pressure in the cerebello-medullary cisterns, pushing and shifting upwards the cerebellar vermis. As a result, the caudal cerebellar folia are compressed and less visible on MRI than the cranial ones. The role of an “intracisternal” obstruction in communicant hydrocephalus with a bulging third ventricle floor has already been hypothesized by Kehler [13] in 2003 and further investigated, with promising results, by Kageyama [12] in 2016 and Al-Hakim [1] in 2019. Venous stenosis, as already theorized by other authors [9, 25], could have a possible role in distal CSF blockade, but this does not explain the good results achieved in our study by ETV in patients with a patent aqueduct.

### Surgical outcome

In our series, ETV showed a satisfactory outcome rate of 66.7%, with a complication rate of 11.1%. Such complications did not require any surgical management. Furthermore, the rate of good outcome could be underestimated, because one of the analyzed patients reported a significant general decline after a prolonged hospitalization for unrelated causes and another one suffered from significant comorbidities, clinical factors which also affected their walking capability and cognitive functions. On the other hand, VPS showed a higher success rate, but with complications reaching 25%.

Although limited, our results appear to be in line with previous studies, such as Ibanez-Botella et al., which report a satisfactory outcome after ventriculostomy in 76% of the cases with a complication rate of 11%. Moreover, Ved et al. claim a 93% success in his ETV cohort with a concomitant 7% complication rate. Other studies about the role of ETV in LOVA consider aqueductal stenosis as an essential requisite for the diagnosis [10] and therefore could not be compared to ours. On the other hand, Kiefer et al. reported a successful outcome in 87% of the cases with a 12% complication rate in a 26 patients cohort treated with gravitational valves in VPS.

**Fig. 3:**
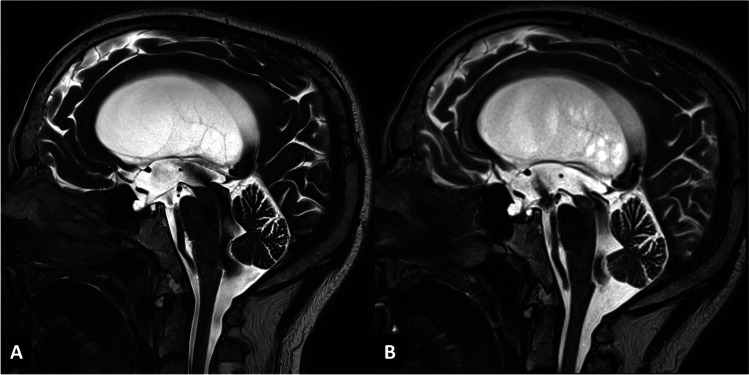
Preoperative and postoperative (4 months of follow-up) T2-weighted MRI in midsagittal view of a LOVA patient from our cohort. Some aforementioned peculiar characteristics are reported, especially a severe ventriculomegaly with an enlarged cisterna magna (**A**). The follow-up imaging shows an adequate CSF flow through the ventriculostomy in the third ventricle floor and a slightly less turbulent flow through the aqueduct, when compared to the preoperative MRI (**B**)

In our opinion, according to our retrospective analysis, ETV remains the first treatment option also in patients with patent aqueduct in the light of an “intracisternal” obstruction hypothesis (Fig. 3). Creating a pathway between the third ventricle and the basal cisterns, according to our opinion, is crucial to bypass the hypothesized intracisternal obstruction in the posterior cerebral fossa distal to the fourth ventricle, with the aim to restore a more physiological CSF flow between the ventricles, the cistern system, and, therefore, the subarachnoid spaces. With the established advances in neuroendoscopy, when performed in a specialized center by an expert surgeon, an ETV procedure carries a relatively low burden of complications, varying from 5 to 15% according to the different clinical series [4]. This was recently confirmed by Jiang et al. in meta-analysis that showed significantly lower complication rate of ETV as compared to VPS [11]. Thus, VPS can be considered a secondary, “rescue” treatment. This is also because, notwithstanding a slightly better chance of successful outcome, VPS has a significantly higher complication rate ranging 17–33%, especially during the first year after the procedure, with a not negligible incidence of CNS infection of 6.1% [11, 17]. VPS for LOVA was also discouraged by Oi et al. because of the risk of overdrainage [23], due to the enormous craniocephalic disproportion and the poor parenchymal compliance due to the longstanding anatomical alteration. We recommend that after ETV, patients with LOVA are followed up by yearly clinical examinations and MRI to evaluate a correct CSF flow through the floor of the third ventricle.

### Limitations

The main limitations of this study are those intrinsic to the retrospective design of the study, including an inevitable selection bias and a not negligible loss to follow-up rate (22.2%). Another source of selection bias could be the exclusion from our cohort of patients with a suspicion and diagnosis of iNPH, by the evaluation of a multidisciplinary board on a clinical and radiological basis. Since the two pathological entities share many traits, although with some suggestive characteristics according to the available literature, such as age, DESH sign, and a narrow callosal angle in one case or cranial circumference and the adherence to Ved’s and Oi’s criteria on the other hand, many patients with LOVA could therefore have been investigated and treated with a suspicion of iNPH and excluded from our study. Moreover, ICP measurement and MR angiography for the evaluation of a potential venous stenosis were not routinely performed in our institution as a part of the diagnostic algorithm. Also, the small sample size due to the uncommon incidence may preclude a more effective statistical analysis and the generalizability of our findings.

## Conclusions and implications of our study

Pathogenesis and optimal surgical treatment of LOVA are disputed in literature, especially in patients with MRI evidence of a patent aqueduct. According to the results of the present study, we hypothesize the presence of intracisternal CSF flow obstruction distally to the fourth ventricle as the etiopathogenetic keystone of open-aqueduct LOVA. Hence, we propose ETV as a first-line treatment in this cohort of patients, relying on its good results, low complication rate, and the aforementioned etiopathogenetic hypothesis.

Nevertheless, a multicentric recruitment of this specific subgroup of patients is necessary to confirm our hypotheses and to establish the best treatment modality. Also, since LOVA shares many characteristics with other types of adulthood hydrocephalus, as previously stated (i.e., LIAS and idiopathic normal pressure hydrocephalus), further studies comparing their clinical and radiological features are warranted.

## Data Availability

The authors declare that the gathered data included and used for analysis outline are available in the manuscript.
